# Factors associated with tuberculosis in persons deprived of liberty in Espírito Santo

**DOI:** 10.11606/s1518-8787.2020054001818

**Published:** 2020-06-26

**Authors:** Laylla Ribeiro Macedo, Ethel Leonor Noia Maciel, Claudio Jose Struchiner

**Affiliations:** I Fundação Oswaldo Cruz Escola Nacional de Saúde Pública Sério Arouca Programa de Pós-graduação em Epidemiologia em Saúde Pública Rio de JaneiroRJ Brasil Fundação Oswaldo Cruz. Escola Nacional de Saúde Pública Sério Arouca. Programa de Pós-graduação em Epidemiologia em Saúde Pública. Rio de Janeiro, RJ, Brasil; II Universidade Federal do Espírito Santo Departamento de Enfermagem VitóriaES Brasil Universidade Federal do Espírito Santo. Departamento de Enfermagem. Vitória, ES, Brasil; III Fundação Oswaldo Cruz Escola Nacional de Saúde Pública Sério Arouca Rio de JaneiroRJ Brasil Fundação Oswaldo Cruz. Escola Nacional de Saúde Pública Sério Arouca. Rio de Janeiro, RJ, Brasil

**Keywords:** Prisoners. Tuberculosis, epidemiology. Tuberculosis, Multidrug-Resistant, epidemiology

## Abstract

**OBJECTIVES:**

To calculate the rate of tuberculosis cases per prison unit in Espírito Santo; present the individual, clinical, and institutional characteristics of the cases in persons deprived of liberty (PPL); and analyze the association between these characteristics and treatment outcome in this population.

**METHODS:**

The study included cases of tuberculosis in the PPL of Espírito Santo from 2014 to 2016. Rate calculation, descriptive analysis and hierarchical logistic regression were performed considering the individual, clinical and institutional levels.

**RESULTS:**

The rate of diagnosed cases per prison unit in the state ranged from 0 to 17.3 cases per 1,000 inmates. Of all reported cases, 218 (72.6%) healed, 21 (7.0%) dropped out, 1 (0.3%) died of tuberculosis, 2 (0.7%) died from other causes, 56 (18.7%) transferred the treatment site and 2 (0.7%) developed drug-resistant tuberculosis. The adjusted analysis showed that supervised treatment ensures success (CR = 0.29; 95%CI 0.01–0.76).

**CONCLUSIONS:**

The study highlighted the importance of knowing the TB treatment outcome in the PPL to implement measures to reduce failure, and the contribution of supervised treatment in this process.

## INTRODUCTION

The prison environment favors disease outbreak in persons deprived of liberty (PPL) — a consequence of confinement and lack of health services access. Besides the diseases already observed in the general population, dermatoses, mental illness, trauma, infectious diarrhea, STDs, and respiratory diseases such as pneumonia and tuberculosis (TB) are often prevalent in PPL^1^.

Monitoring TB indicators in the PPL of Brazil showed an increase in the incidence rate from 627.6 cases to 904.9 cases per 100,000 prisoners from 2007 to 2013, while mortality reached 18.0 deaths per 100,000 prisoners in 2007 and 16.0 deaths per 100,000 prisoners in 2013^2^. Some factors effectively contribute to the PPL high endemicity of tuberculosis. They may be related to individuals and their living conditions before incarceration — being young, male and with low education; using drugs; having associated injuries; exhibiting previous treatment for TB and having a history of incarceration; or to incarceration — overpopulation, poorly ventilated and poorly lit cells, frequent exposure to the bacillus in a confined environment, lack of information and lack of health services access in prison^3^.

In 2014, the Ministry of Health launched the *Política Nacional de Atenção Integral à Saúde das Pessoas Privadas de Liberdade no Sistema Prisional* (PNAISP – National Policy for Comprehensive Health Care for People Deprived of Liberty in the Prison System), instituted by Interministerial Ordinance no. 1, of January 2, 2014, to expand the health measures of the Brazilian Unified Health System (SUS) for PPL. Among its multiple guidelines, PNAISP also included a specific measure for TB control^4^.

Thus, besides quantifying TB cases in PPL, we must investigate which factors permeate this context and interfere in the dynamics of disease outbreak and treatment. This study aimed to calculate the rate of tuberculosis cases per prison unit in Espírito Santo (ES) from 2014 to 2016; to present the individual, clinical, and institutional characteristics of the cases; and to analyze the association between these characteristics and the interruption of tuberculosis treatment in this population.

## METHODS

The sample of this study comprised the tuberculosis cases registered in persons deprived of liberty in the State of Espírito Santo from 2014 to 2016, including new cases, relapses, readmission after dropping out, transfer or unknown entry method (“does not know”). Espírito Santo has 34 prison units, which in 2014 housed 16,234 prisoners, accounting for an imprisonment rate of 417.9 PPL per 100,000 inhabitants, higher than the country’s rate—299.7 per 100,000^5^. These units refer to provisional, closed, semi-open and mixed prison systems, excluding police stations or the like.

**Figure 1:**
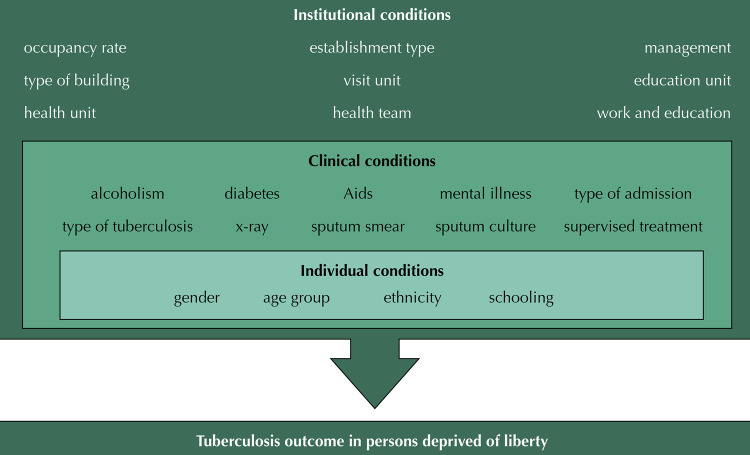
Hierarchical model of the relationship between incarceration and tuberculosis outcome. PRBSF: Penitenciária Regional Barra de São Francisco; CPFCI: Centro Prisional Feminino de Cachoeiro de Itapemirim; HCTP: Hospital de Custódia e Tratamento Psiquiátrico; PSMCOL: Penitenciária de Semiaberta Masculina de Colatina; CPFCOL: Centro Prisional Feminino de Colatina; CDPSM: Centro de Detenção Provisória de São Mateus; CDPFV: Centro de Detenção Provisória Feminino de Viana; PRL: Penitenciária Regional de Linhares; PRSM: Penitenciária Regional de São Mateus; PFC: Penitenciária Feminina de Cariacica; CDPA: Centro de Detenção Provisória de Aracruz; PEVV: Penitenciária Estadual de Vila Velha (1, 2, 3 e 5); PSMECOL: Penitenciária de Segurança Média de Colatina; CDPS: Centro de Detenção Provisória da Serra; PSVV: Penitenciaria Semiaberta de Vila Velha; PSMA: Penitenciária de Segurança Máxima (1 e 2); PRCI: Penitenciária Regional de Cachoeiro de Itapemirim; CDPCI: Centro de Detenção Provisória de Cachoeiro de Itapemirim; CASCUVV: Casa de Custódia de Vila Velha; PAES: Penitenciária Agrícola do Espirito Santo; CDPCOL: Centro de Detenção Provisória de Colatina; CDPSDN: Centro de Detenção Provisória de São Domingos do Norte; CDPV 2: Centro de Detenção Provisória de Viana 2; PSME 1: Penitenciária de Segurança Média 1; PSC: Penitenciária Semiaberta de Cariacica; CDPG: Centro de Detenção Provisória de Guarapari; CDPVV: Centro de Detenção Provisória de Vila Velha; CDPM: Centro de Detenção Provisória de Marataízes; CDRL: Centro de Detenção Provisória de Linhares; CTV: Centro de Triagem de Viana

The study variables were categorized into hierarchical levels based on the models proposed by Maciel^6^ and the literature on the subject. Thus, the theoretical model used consisted of the levels and respective variables (Figure 1).

The variable studied was the tuberculosis treatment outcome, classified by the *Programa Nacional de Controle da Tuberculose* (National Tuberculosis Control Program) as: cure, dropping out, death from tuberculosis, death from other causes, transfer, multidrug-resistant tuberculosis, treatment change, failure, and primary default. In the hierarchical logistic model, this variable was reclassified as success (cure) and failure (other categories).

Data were collected from the *Sistema de Informação de Agravos de Notificação* (SINAN – Health Information System) and the *Levantamento Nacional de Informações Penitenciárias*^5^(Infopen – National Penitentiary Information Survey), as well as databases and reports from the State health and justice departments, stored in a single database for statistical analysis.

Statistical analysis estimated the TB diagnosis rate per prison unit in Espírito Santo, considering only the cases diagnosed during incarceration, and excluding transfers and unknown entry method (“does not know”). Then a descriptive analysis was performed according to the variables ranked by level and categorized per TB treatment outcome.

Logistic regression estimated the gross and adjusted odds ratio (OR) and respective 95% confidence intervals (95%CI). Multivariate analysis used the hierarchical model defined according to the established levels. Initially, the variables associated with the interest outcome (p ≤ 0.20) were included in the model, to account for possible confounding variables. In the modeling stage, independent variables were included from the distal to the proximal level. Variables with p < 0.20 were kept at each hierarchical level and in the final model for adjustment. In the final model, a significance level of 5% was adopted to determine the variables associated with treatment failure.

Hierarchical models are used when distal factors influence intermediate factors, which, in turn, influence more proximal factors, directly impacting the outcome. Thus, this model allows including variables in their sequential order, even with dependency on observations^6^. Statistical analyses were performed using the R programming, version 3.4.1.

This research was approved by the Research Ethics Committee of the Escola Nacional de Saúde Pública Sérgio Arouca da Fundação Oswaldo Cruz (Fiocruz) under opinion no. 1,866,469 on December 14, 2016.

## RESULTS

**Figure 2:**
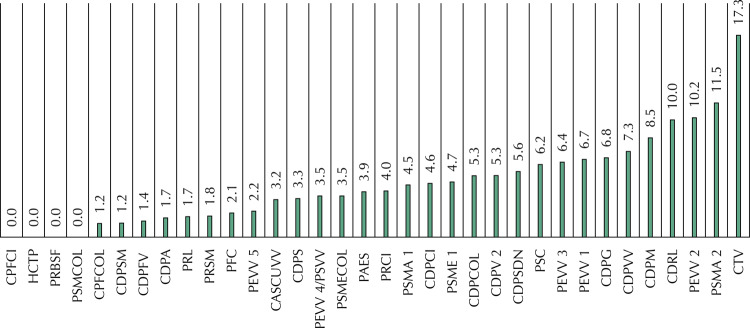
Rate of tuberculosis cases per 1,000 prisoners in each prison unit. Espírito Santo, 2014–2016.

The rate of tuberculosis cases diagnosed in Espírito Santo’s prison units from 2014 to 2016 ranged from 0 to 17.3 cases per 1,000 prisoners. Units with no TB cases were mostly from the countryside and had an occupancy rate below 100%; the *Centro de Triagem de Viana* (CTV – Viana Triage Center), located in Grande Vitória, had the highest diagnosis rate and an occupancy rate above 200%. Other institutional characteristics proved to be heterogeneous among the units (Figure 2).

According to SINAN, in 2014, 2015 and 2016 the PPL of Espírito Santo registered 103, 91 and 106 cases of tuberculosis, respectively, totaling 300 cases. Of these, 218 (72.6%) healed, 21 (7.0%) dropped out, 1 (0.3%) died of tuberculosis, 2 (0.7%) died from other causes, 56 (18.7%) transferred the treatment site and 2 (0.7%) developed drug-resistant TB.

We evaluated these cases for their individual, clinical and institutional characteristics (Table 1) and categorized them according to treatment outcome (success and failure).

**Table 1:** Individual and clinical characteristics of tuberculosis cases in persons deprived of liberty in Espírito Santo, from 2014 to 2016, according to treatment outcome (n = 300).

Characteristics	Success n (%)	Failure n (%)	Total cases n (%)
Individual conditions			
Gender			
Male	216 (73.7)	77 (26.3)	293 (97.7)
Female	2 (28.6)	5 (71.4)	7 (2.3)
Age group			
18 to 29 years	144 (75.0)	48 (25.0)	192 (64.0)
30 to 39 years old	53 (66.2)	27 (33.8)	80 (26.7)
40 years or older	21 (75.0)	7 (25.0)	28 (9.3)
Ethnicity			
White	46 (65.7)	24 (34.3)	70 (23.3)
Black	33 (70.2)	14 (29.8)	47 (15.7)
Asian	1 (100.0)	0 (0.0)	1 (0.3)
Mixed race	127 (76.5)	39 (23.5)	166 (55.4)
Indigenous	0 (0.0)	0 (0.0)	0 (0.0)
Not reported	11 (68.8)	5 (31.2)	16 (5.3)
Schooling level			
Illiterate	6 (85.7)	1 (14.3)	7 (2.3)
1 to 4 years	35 (56.7)	27 (43.5)	62 (20.7)
5 to 8 years	109 (83.8)	21 (16.2)	130 (43.3)
> 8 years	41 (78.8)	11 (21.2)	52 (17.3)
Not applicable	1 (50.0)	1 (50.0)	2 (0.7)
Not reported	26 (55.3)	21 (44.7)	47 (15.7)
Clinical conditions			
Aids			
No	193 (78.1)	54 (21.9)	247 (82.3)
Yes	11 (61.1)	7 (38.9)	18 (6.0)
Not reported	14 (40.0)	21 (60.0)	35 (11.7)
Diabetes mellitus			
No	207 (74.2)	72 (25.8)	279 (93.0)
Yes	5 (83.3)	1 (16.7)	6 (2.0)
Not reported	6 (40.0)	9 (60.0)	15 (5.0)
Mental illness			
No	213 (74.2)	74 (25.8)	287 (95.7)
Yes	2 (50.0)	2 (50.0)	4 (1.3)
Not reported	3 (33.3)	6 (66.7)	9 (3.0)
Alcoholism			
No	164 (74.2)	57 (25.8)	221 (73.7)
Yes	52 (72.2)	20 (27.8)	72 (24.0)
Not reported	2 (28.6)	5 (71.4)	7 (2.3)
Admission			
New case	159 (74.0)	56 (26.0)	215 (71.7)
Relapse	15 (65.2)	8 (34.8)	23 (7.7)
Re-entry after dropping out	25 (75.8)	8 (24.2)	33 (11.0)
Does not know	1 (100.0)	0 (0.0)	1 (0.3)
Transfer	18 (64.3)	10 (35.7)	28 (9.3)
Postmortem	0 (0.0)	0 (0.0)	0 (0.0)
Type			
Pulmonary	197 (74.3)	68 (25.7)	265 (88.3)
Extrapulmonary	16 (59.3)	11 (40.7)	27 (9.0)
Pulmonary + extrapulmonary	5 (62.5)	3 (37.5)	8 (2.7)
Radiography			
Normal	6 (66.7)	3 (33.3)	9 (3.0)
Suspect	183 (75.0)	61 (25.0)	244 (81.3)
Other pathology	2 (40.0)	3 (60.0)	5 (1.7)
Not performed	20 (60.6)	13 (39.4)	33 (11.0)
Not reported	7 (77.8)	2 (22.2)	9 (3.0)
Sputum smear			
Negative	56 (77.8)	16 (22.2)	72 (24.0)
Positive	150 (72.5)	57 (27.5)	207 (69.0)
Not performed	10 (62.5)	6 (37.5)	16 (5.3)
Not applicable	2 (40.0)	3 (60.0)	5 (1.7)
Sputum culture			
Negative	37 (71.2)	15 (28.8)	52 (17.3)
Positive	74 (81.3)	17 (18.7)	91 (30.3)
Pending	57 (64.0)	32 (36.0)	89 (29.7)
Not performed	50 (73.5)	18 (23.5)	68 (22.7)
Supervised treatment			
No	21 (58.3)	15 (41.7)	36 (12.0)
Yes	191 (84.9)	34 (15.1)	225 (75.0)
Not reported	6 (15.4)	33 (84.6)	39 (13.0)

The cases were mostly male, young (18 to 29 years), mixed race, with five to eight years of schooling. Women and individuals aged 30 to 39 years, with one to four years of schooling, showed more treatment failure.

Among comorbidities and associated conditions, the most prevalent was alcoholism, reported in 24% of cases, followed by Aids (6.0%), diabetes (2.0%) and mental illness (1.2%). Results show that most treatment failures occurred in cases with Aids and mental illness. The percentage of cure (success) was similar between alcohol users (72.2%) and non-users (74.2%).

The most observed types of admission were new case (71.7%) and pulmonary tuberculosis (88.3%). Cases of extrapulmonary TB showed less treatment success when compared to pulmonary TB.

Most X-ray examinations were suspected, while sputum smear and sputum culture tests were positive. Those treated with medication showed a success rate above 80%, approaching 60% in cases without the supervised dose.

Regarding institutional aspects, most cases occurred in provisional units, managed by the state, with an occupancy rate between 100.00 and 199.99%. The infrastructure assessment showed the prevalence of cases in institutions built for incarceration, with an expanded health (74.7%), education (70.0%) and visit (46.0%) structure. We also highlight the presence of a differentiated expanded health team, comprising a nursing team, general practitioner, dentist, social worker and psychologist or occupational therapist, or psychiatrist, or physiotherapist for 63.0% of cases. The work rate was 0.10 to 14.99% in the prison units where 65.6% of TB cases were diagnosed, and the most prevalent education rate was 15.00 to 29.99%, representing 32.3% of them. Table 2 details these data by treatment outcome (success and failure).

**Table 2:** Institutional characteristics of the tuberculosis cases in persons deprived of liberty in Espírito Santo, from 2014 to 2016, according to treatment outcome ().

Characteristics	Success n (%)	Failure n (%)	Total of cases n (%)
Institutional Conditions			
Establishment type			
Provisional	98 (71.0)	40 (29.0)	138 (46.0)
Closed	94 (82.5)	20 (17.5)	114 (38.0)
Semi-open	17 (50.0)	17 (50.0)	34 (11.3)
Mixed	9 (64.3)	5 (35.7)	14 (4.7)
Safety measure	0 (0.0)	0 (0.0)	0 (0.0)
Occupation rate			
Below 99.99%	10 (58.8)	7 (41.2)	17 (5.7)
100.00–199.99%	180 (72.6)	68 (27.4)	248 (82.6)
200.00% or more	28 (80.0)	7 (20.0)	35 (11.7)
Management			
Public	207 (71.9)	81 (28.1)	288 (96.0)
Co-management	11 (91.7)	1 (8.3)	12 (4.0)
Type of building			
Designed	197 (72.2)	76 (27.8)	273 (91.0)
Adapted	21 (77.8)	6 (22.2)	27 (9.0)
Visit unit			
No	27 (71.1)	11 (28.9)	38 (12.7)
Minimum	84 (67.7)	40 (32.3)	124 (41.3)
Expanded	107 (77.5)	31 (22.5)	138 (46.0)
Health unit			
Support	6 (66.7)	3 (33.3)	9 (3.0)
Minimum	33 (67.3)	16 (32.7)	49 (16.3)
Differentiated minimum	13 (72.2)	5 (27.8)	18 (6.0)
Expanded	166 (74.1)	58 (25.9)	224 (74.7)
Education unit			
No	49 (73.1)	18 (26.9)	67 (22.3)
Minimum	18 (78.3)	5 (21.7)	23 (7.7)
Expanded	151 (71.9)	59 (28.1)	210 (70.0)
Health team			
Technical only	10 (52.6)	9 (47.4)	19 (6.3)
Minimum	20 (76.9)	6 (23.1)	26 (8.7)
Differentiated minimum	35 (70.0)	15 (30.0)	50 (16.7)
Expanded	8 (50.0)	8 (50.0)	16 (5.3)
Differentiated expanded	145 (76.7)	44 (23.3)	189 (63.0)
Work rate			
No	30 (78.9)	8 (21.1)	38 (12.7)
0.10–14.99%	142 (72.1)	55 (27.9)	197 (65.6)
15.00–29.99%	35 (89.7)	4 (10.3)	39 (13.0)
30.00–44.99%	3 (27.3)	8 (72.7)	11 (3.7)
45.00–59.99%	3 (60.0)	2 (40.0)	5 (1.7)
60.00% or more	5 (50.0)	5 (50.0)	10 (3.3)
Education rate			
No	51 (70.8)	21 (29.2)	72 (24.0)
0.10–14.99%	61 (65.6)	32 (34.4)	93 (31.0)
15.00–29.99%	77 (79.4)	20 (20.6)	97 (32.3)
30.00–44.99%	9 (81.8)	2 (18.2)	11 (3.7)
45.00–59.99%	11 (64.7)	6 (35.3)	17 (5.7)
60.00% or more	9 (90.0)	1 (10.0)	10 (3.3)

Table 3 shows the characteristics that remained in the final model. The adjusted analysis showed that supervised treatment for tuberculosis prevents treatment failure (CR = 0.29; 95%CI 0.01–0.76).

**Table 3:** Gross and adjusted analysis (hierarchical) of tuberculosis cases in persons deprived of liberty in Espírito Santo, from 2014 to 2016, according to treatment outcome.

Characteristics	Gross OR (95%CI)	p-value	Adjusted OR (95%CI)	p-value
				
Aids		0.105		0.159
No	1.00		1.00	
Yes	2.27 (0.80–6.06)		2.26 (0.59–8.62)	
Type		0.112		0.044
Pulmonary	1.00		1.00	
Extrapulmonary	1.99 (0.86–4.46)		1.81 (0.38–8.52)	
Pulmonary + extrapulmonary	1.73 (0.34–7.27)		4.98 (0.51–48.11)	
Sputum smear		0.058		0.094
Negative	1.00		1.00	
Positive	1.33 (0.71–2.56)		1.31 (0.42–4.40)	
Not performed	2.10 (0.63–6.59)		2.19 (0.54–16.19)	
Not applicable	5.25 (0.80–42.54)		43.85 (1.47–1303.20)	
Radiography		0.084		0.220
Normal	1.00		1.00	
Suspect	0.66 (0.17–3.23)		0.38 (0.04–3.08)	
Other pathology	3.00 (0.32–34.76)		1.29 (0.07–22.38)	
Not performed	1.30 (0.28–7.02)		0.92 (0.09–8.93)	
Supervised treatment		< 0.001		0.002
No	1.00		1.00	
Yes	0.24 (0.11–0.53)		0.29 (0.10–0.76)	
Management		0.165		0.123
Public	1.00		1.00	
Co-management	0.23 (0.01–1.22)		0.21 (0.26–1.70)	
Occupancy rate		0.124		0.037
below 99.99%	1.00		1.00	
100.00–199.99%	0.53 (0.19–1.53)		0.51 (0.18–1.43)	
200.00% or more	0.35 (0.09–1.27)		0.23 (0.06–0.88)	
Visit unit		0.170		0.040
No	1.00		1.00	
Minimum	1.16 (0.53–2.67)		1.03 (0.45–2.33)	
Expanded	0.71 (0.32–164)		0.52 (0.22–1.23)	

OR: odds ratio; 95%CI: 95% confidence interval.

## DISCUSSION

This study had some limitations. It left out variables on time of incarceration during diagnosis or related to previous history of incarceration, which could influence diagnosis, although there are still little data showing any association with treatment outcome. The impact of TB on persons deprived of liberty in the general burden of the disease may be underestimated when considering the situations in which the PPL develop it after release, albeit acquiring the bacillus during incarceration. Thus, other studies should address these questions.

In the period studied, the CTV had the highest rate of TB diagnosed cases, with 17.3 per 1,000 persons deprived of liberty. The CTV is singular if compared with other institutions, working as a gateway to the ES prison system and therefore has a high turnover, with an average of 690 entries each month^7^. The established flow foresees that these newcomers remain for a maximum period of 48 hours in the unit, awaiting transfer according to profile and legal situation, identified by an initial registration. However, this deadline is not met, with prisoners remaining for up to 30 days^8^.

According to the *Protocolo de Controle de Tuberculose Pulmonar da População Prisional* (Protocol for Controlling Pulmonary Tuberculosis in the Prison Population) of Espírito Santo based on the Ministry of Health guidelines, an active search for respiratory symptoms, using a specific screening form, must be performed when entering the prison system^9^—which may be responsible for the greater number of TB cases identified in the CTV compared with the other units. Once transferred, these individuals already have an initial screening for TB. However, although the diagnosis occurs during incarceration, we must reflect on the transmission mechanism of the TB bacillus and its incubation time. From our data, it is unclear if the prison unit was the place of TB contagion, since the PPL could already be infected, progressing to the active disease during this period. Although not the source, the prison environment would have contributed to the disease evolution^3^. Genetic studies could provide a better understanding on the dynamics of *M. tuberculosis* transmission, enlightening this issue. Knowledge of how the microorganism is dispersed in the prison and general population aids in disease prevention and control strategies^10^.

Tuberculosis cases recorded in ES prison population in 2014, 2015 and 2016 represented 7.5%, 6.1% and 7.8% of all reported cases, respectively. In 2014, 8.4% of all cases in Brazil were found among PPL, although this group represented only 0.3% of the country’s total population in that year^11^.

According to the Ministry of Health, comparative data on TB treatment outcome in 2015 showed cure, dropping out, and transfer rates of 73.2%, 7.7% and 5.5% for PPL, while in the general population they reached 71.0%, 9.8% and 5.0%, respectively^12^. Our data on cure (72.6%) and dropping out (7.0%) rates were similar to this study, but the transfer rate (18.7%) was superior, probably due to the logistics adopted by the ES Department of Justice, which elected one prison unit as the gateway to the prison system, from which the allocation is made.

Frequent transfers between units influence follow-up loss; thus, communication between safety and health professionals must be agile and constant, aiming to minimize treatment drop out and ensuring that the medical records accompany the patient when changing prisons. The Ministry of Health states that mobility in the prison system increases the risk of TB infection, given the circulation between different penal institutions, health services and the general population, during and after incarceration^13^. Although the rate of incarcerated women increased by 10.7% per year from 2005 to 2014, men are still most of PPL, with an average of 5.8% women to 94.2% men^5^. In Espírito Santo, men represented 93.6% of the all arrests in 2014^5^, supporting the high percentage of TB cases in men observed in this study. Despite being a minority, the research showed a higher proportion of treatment failure in women (71.4%) than in men (26.3%). A study assessing TB in the Brazilian PPL from 2009 to 2014^11^ showed that women deprived of liberty had HIV co-infection rates (24.1%) higher than men (15.2%). In this study, co-infection appeared in 28.6% of women and 5.5% of men, and may contribute significantly to the observed failure scenario, though associated with other characteristics.

Infopen data show that approximately 55% of the Brazilian PPL is between 18 and 29 years of age^6^, age group of most TB cases recorded in the study (64%). A wider age group, from 18 to 39 years, accounts for 90.7% of the recorded cases. Previous studies also show that the age group 18 to 39 years (74.7%) comprises most TB cases recorded in the PPL, although only 39.8% of TB cases in the general population show up in this age range^11^. In 2014, 25.6% of TB cases in men aged 20 to 29 years registered in Brazil were deprived of liberty^11^.

Extrapulmonary tuberculosis rate, observed in 9.0% of all cases in the PPL, was similar to the country’s, 8.2%^11^. The results showed a higher percentage of treatment success for pulmonary TB (74.3%) than for extrapulmonary (59.3%). Extrapulmonary TB diagnosis presents a higher degree of difficulty, since some forms show unspecific signs and symptoms, requiring tests of restricted access to PPL and delaying treatment, which may explain this finding^13^.

The institutional description observed refers to the characteristics of the prison units where TB cases were located during diagnosis and reflects the prison context experienced in the state in general. In 2016, the occupancy rate of prisons in the country was 197.4% and 144.7% in Espírito Santo, while the rate of prisoners without charge in Brazil was 40.2% and 42.3% in the ES^14^, according to the literature.

In 2006, the ES prison system underwent overpopulation, violent rebellions, homicides, and breakouts, resulting in the absence of a minimum structure that could enable security management or resocialization policy. Thus, in 2009, a federal intervention carried out by the *Conselho Nacional de Política Criminal e Penitenciária* (National Council for Criminal and Penitentiary Policy), restructured the ES prison system by building 26 prison units, which included infrastructure for visit, education, work, and health. State management aimed to implement policies in security and resocialization, including health care, formal study provision, training and work^15^. The ES currently has work and education rates among the best recorded in the country^15,14,16^. Building ventilated and illuminated prisons contributes to reducing the transmission of respiratory infections, according to the *Manual de Intervenções Ambientais para o Controle da Tuberculose nas Prisões* (Environmental Interventions Handbook for Controlling Tuberculosis in Prisons), which proposes solutions to improve environmental conditions for preserving peoples’ health without compromising safety^17^.

Regarding health, the coverage of the *Programa de Saúde Prisional* (Prison Health Program) in ES was 75.2% in 2014^18^, reaching 100.0% in 2018. This study highlighted the importance of the health team’s performance for treatment success. The PNAISP, established by the Ministry of Health in 2014, aims to ensure access for persons deprived of liberty to comprehensive care in the SUS, focus of the *Rede de Atenção à Saúde* (RAS – Health Care Network). This national policy and its ordinances standardize the types of teams and resource funding. The *Equipe de Atenção Básica Prisional* (EABp – Prison Primary Care Team) is multidisciplinary, and its modality and workload are determined by the number of inmates and the epidemiological profile of the units^4^.

Performing directly observed treatment (DOT) was associated with treatment success, reaffirming the beneficial effect of DOT shown in other studies^19^. One research showed that supervised treatment reduces unfavorable tuberculosis treatment outcomes by 25%, and that patients who did not receive DOT were more likely to abandon treatment (OR = 0.62; 95%CI 0.57–0.66), die from TB (OR = 0.68; 95%CI 0.61–0.77) and have unknown treatment results (OR = 0.71; 95%CI 0.66–0.76)^20^. The Ministry of Health recommends directly observed treatment for PPL, as it strengthens the bond with the health professional, guarantees quick access to health service regarding adverse effects, prevents using medication as an element of exchange and pressure, offers to exchange information and establish care, among others^13^.

The ministry also lists as main obstacles to implementing control strategies in the prison environment: the lack of knowledge about TB by PPL and security professionals; undervaluation of TB symptoms by PPL, hindering early diagnosis; difficulty of PPL access to health services, and its scarcity of human and financial resources; low adherence of PPL to treatment and prevention measures; and the stigmatization and segregation created by TB, considering the importance of protection by belonging to a group^13^.

Despite the challenges, the study showed that we must examine the components influencing the outcome of tuberculosis treatment in PPL, to implement measures that provide cure and reduce failure rates. Knowing what leads to drop out contributes to interrupt the chain of transmission of the disease and its prevention, not only among the PPL, but between family members and workers who circulate in the prison environment.
