# Supplementary material for: Factors associated with tuberculosis in persons deprived of liberty in Espírito Santo
Source: Rev Saude Publica. 2020 Jun 26;54:67. doi: 10.11606/s1518-8787.2020054001818 (PMC7319498; doi:10.11606/s1518-8787.2020054001818)
Supplement: Translation

# Translation: Fatores associados à tuberculose na população privada de liberdade no Espírito Santo

**Authors:** Laylla Ribeiro Macedo, Ethel Leonor Noia Maciel, Claudio Jose Struchiner

**Affiliations:** I Brasil Fundação Oswaldo Cruz. Escola Nacional de Saúde Pública Sério Arouca. Programa de Pós-graduação em Epidemiologia em Saúde Pública. Rio de Janeiro, RJ, Brasil; II Brasil Universidade Federal do Espírito Santo. Departamento de Enfermagem. Vitória, ES, Brasil; III Brasil Fundação Oswaldo Cruz. Escola Nacional de Saúde Pública Sério Arouca. Rio de Janeiro, RJ, Brasil

**Keywords:** Prisioneiros, Tuberculose, epidemiologia, Tuberculose Resistente a Múltiplos Medicamentos, epidemiologia

## Abstract

**OBJETIVOS:**

Calcular a taxa de casos diagnosticados com tuberculose por unidades prisionais do Espírito Santo, apresentar as características individuais, clínicas e institucionais dos casos na população privada de liberdade (PPL) do ES e analisar a associação entre essas características e o encerramento do tratamento da tuberculose nessa população.

**MÉTODOS:**

A população de estudo foram os casos de tuberculose na PPL do ES de 2014 a 2016. Realizou-se o cálculo de taxa, a análise descritiva e a regressão logística hierarquizada considerando os níveis individual, clínico e institucional.

**RESULTADOS:**

A taxa de casos diagnosticados por unidade prisional no estado variou de 0 a 17,3 casos por 1.000 presos. Do total de casos notificados, 218 (72,6%) se curaram, 21 (7,0%) abandonaram o tratamento, 1 (0,3%) morreu por tuberculose, 2 (0,7%) morreram por outras causas, 56 (18,7%) transferiram o local de tratamento e 2 (0,7%) desenvolveram tuberculose drogarresistente. A análise ajustada mostrou que o tratamento supervisionado é um fator protetor para o insucesso (RC = 0,29; IC95% 0,01–0,76).

**CONCLUSÕES:**

O estudo apontou a importância do conhecimento do desfecho do tratamento da TB na PPL visando à implementação de ações para a redução do insucesso, bem como a contribuição do tratamento supervisionado nesse processo.

## INTRODUÇÃO

O ambiente prisional favorece o adoecimento da população privada de liberdade (PPL) devido às consequências do confinamento e da dificuldade de acesso aos serviços de saúde. Além dos agravos já observados na população em geral, são frequentemente prevalentes na PPL dermatoses, transtornos mentais, traumas, diarreias infecciosas, doenças sexualmente transmissíveis e ainda doenças respiratórias como pneumonia e tuberculose (TB), principalmente^1^.

O monitoramento dos indicadores da TB na PPL no Brasil mostrou um aumento na taxa de incidência de 627,6 casos para 904,9 casos por 100 mil presos de 2007 para 2013, enquanto a taxa de mortalidade nesse período foi de 18,0 óbitos por 100 mil presos em 2007 e 16,0 óbitos por 100 mil presos em 2013^2^. Alguns fatores contribuem efetivamente para alta endemicidade da tuberculose na PPL. Eles podem ser atribuídos aos indivíduos e sua condição de vida antes do encarceramento, como ser jovem, do sexo masculino e com baixa escolaridade, fazer uso de drogas, apresentar agravos associados, ter feito tratamento anterior para TB e ter antecedente de encarceramento; ou ainda aos fatores relacionados ao encarceramento, como celas superpopulosas, mal ventiladas e com pouca iluminação solar, exposição frequente ao bacilo em ambiente confinado, falta de informação e dificuldade de acesso aos serviços de saúde na prisão^3^.

Em 2014, o Ministério da Saúde (MS) lançou a Política Nacional de Atenção Integral à Saúde das Pessoas Privadas de Liberdade no Sistema Prisional (PNAISP), instituída pela portaria interministerial nº 1, de 2 de janeiro de 2014, com o objetivo de ampliar as ações de saúde do Sistema Único de Saúde (SUS) para a PPL. Entre as múltiplas diretrizes do PNAISP, também foi incorporada uma linha de ação específica para o controle da TB^4^.

Diante disso, é de extrema importância, além de quantificar os casos de TB na PPL, conhecer os fatores que permeiam esse contexto e que interferem na dinâmica do adoecimento e tratamento da doença. Os objetivos deste estudo foram: calcular a taxa de casos diagnosticados com tuberculose por unidades prisionais do Espírito Santo no período de 2014 a 2016, apresentar as características individuais, clínicas e institucionais dos casos de TB na PPL do ES no período de 2014 a 2016 e analisar a associação entre essas características e a situação de encerramento do tratamento da tuberculose na PPL do ES.

## MÉTODOS

A população total do estudo foram os casos de tuberculose registrados na PPL do ES de 2014 a 2016, incluindo casos novos, recidivas, reingressos após abandono, transferência ou método de entrada desconhecido (“não sabe”). O ES possui 34 unidades prisionais (UP), que em 2014 abrigavam 16.234 presos, contabilizando uma taxa de aprisionamento de 417,9 pessoas privadas de liberdade por 100 mil habitantes, superior à taxa do Brasil, que é de 299,7 por 100 mil^5^. Essas UP referem-se a estabelecimentos de regime do tipo provisório, fechado, semiaberto e misto, sendo excluídas delegacias ou similares.

As variáveis do estudo foram categorizadas em níveis hierarquizados construídos baseando-se nos modelos propostos por Maciel^6^ e na literatura acerca do tema. Sendo assim, o modelo teórico utilizado foi constituído pelos níveis e respectivas variáveis, conforme a Figura 1.

**Figura 1:** Modelo hierarquizado da relação entre encarceramento e desfecho da tuberculose.

A variável de desfecho estudada foi a situação de encerramento do tratamento da tuberculose, classificada pelo Programa Nacional de Controle da Tuberculose como cura, abandono, óbito por tuberculose, óbito por outras causas, transferência, tuberculose multidrogarresistente, mudança de esquema, falência e abandono primário. No modelo logístico hierarquizado, essa variável foi reclassificada em sucesso (cura) e insucesso (demais categorias).

Os dados utilizados foram extraídos do Sistema de Informação de Agravos de Notificação (SINAN) e do Levantamento Nacional de Informações Penitenciárias^5^, além de bancos de dados e relatórios das secretarias de saúde e de justiça do ES, armazenados em uma base de dados única para análise estatística.

Na análise estatística foi realizado o cálculo das taxas de diagnóstico com TB por unidade prisional do ES. Para esse cálculo foram considerados somente os casos diagnosticados durante o encarceramento, excluindo-se os casos de transferências e método de entrada desconhecido (“não sabe”). Em seguida realizou-se a análise descritiva dos casos na PPL do ES, segundo as variáveis estabelecidas nos níveis e categorizadas de acordo com a situação de encerramento da TB.

A regressão logística foi utilizada para calcular as razões de chances (RC) brutas e ajustadas e respectivos intervalos de confiança de 95% (IC95%). Na análise multivariável, foi empregado o modelo hierarquizado definido de acordo com os níveis estabelecidos. Inicialmente, as variáveis associadas com o desfecho de interesse (p ≤ 0,20) foram incluídas no modelo, a fim de considerar a inclusão de possíveis variáveis de confusão. Na etapa de modelagem, as variáveis independentes foram incluídas a partir do nível distal até o proximal. Foram mantidas em cada nível hierarquizado e no modelo ﬁnal as variáveis com p < 0,20 para ajuste. No modelo final, adotou-se o nível de significância de 5% para determinação das variáveis associadas ao insucesso do tratamento.

Modelos hierarquizados são utilizados quando fatores distais influenciam fatores intermediários, que, por sua vez, influenciam fatores mais proximais, agindo diretamente no desfecho. Dessa maneira, este modelo permite incluir variáveis de acordo com sua ordenação temporal, mesmo que haja dependência das observações^6^. As análises estatísticas foram realizadas no programa R na versão 3.4.1.

Esta pesquisa foi aprovada pelo Comitê de Ética em Pesquisa da Escola Nacional de Saúde Pública Sérgio Arouca da Fundação Oswaldo Cruz (Fiocruz) sob o parecer nº 1.866.469 em 14 de dezembro de 2016.

## RESULTADOS

A taxa de casos de tuberculose diagnosticados nas unidades prisionais do ES de 2014 a 2016 variou de 0 a 17,3 casos por 1.000 presos. As unidades que não contabilizaram casos de TB eram em sua maioria localizadas em cidades do interior do estado e tinham taxa de ocupação inferior a 100%, enquanto o Centro de Triagem de Viana (CTV), que apresentou a maior taxa de diagnóstico, localiza-se na Grande Vitória e tinha taxa de ocupação acima de 200%. Outras características institucionais mostraram-se heterogêneas entre as unidades (Figura 2).

**Figura 2:** Taxa de casos diagnosticados com tuberculose por 1.000 presos em cada unidade prisional. Espírito Santo, 2014–2016. PRBSF: Penitenciária Regional Barra de São Francisco; CPFCI: Centro Prisional Feminino de Cachoeiro de Itapemirim; HCTP: Hospital de Custódia e Tratamento Psiquiátrico; PSMCOL: Penitenciária de Semiaberta Masculina de Colatina; CPFCOL: Centro Prisional Feminino de Colatina; CDPSM: Centro de Detenção Provisória de São Mateus; CDPFV: Centro de Detenção Provisória Feminino de Viana; PRL: Penitenciária Regional de Linhares; PRSM: Penitenciária Regional de São Mateus; PFC: Penitenciária Feminina de Cariacica; CDPA: Centro de Detenção Provisória de Aracruz; PEVV: Penitenciária Estadual de Vila Velha (1, 2, 3 e 5); PSMECOL: Penitenciária de Segurança Média de Colatina; CDPS: Centro de Detenção Provisória da Serra; PSVV: Penitenciaria Semiaberta de Vila Velha; PSMA: Penitenciária de Segurança Máxima (1 e 2); PRCI: Penitenciária Regional de Cachoeiro de Itapemirim; CDPCI: Centro de Detenção Provisória de Cachoeiro de Itapemirim; CASCUVV: Casa de Custódia de Vila Velha; PAES: Penitenciária Agrícola do Espirito Santo; CDPCOL: Centro de Detenção Provisória de Colatina; CDPSDN: Centro de Detenção Provisória de São Domingos do Norte; CDPV 2: Centro de Detenção Provisória de Viana 2; PSME 1: Penitenciária de Segurança Média 1; PSC: Penitenciária Semiaberta de Cariacica; CDPG: Centro de Detenção Provisória de Guarapari; CDPVV: Centro de Detenção Provisória de Vila Velha; CDPM: Centro de Detenção Provisória de Marataízes; CDRL: Centro de Detenção Provisória de Linhares; CTV: Centro de Triagem de Viana

Nos anos de 2014, 2015 e 2016, foram notificados no SINAN respectivamente 103, 91 e 106 casos de tuberculose na PPL do ES, totalizando 300 casos no período. Do total de casos na PPL, 218 (72,6%) se curaram, 21 (7,0%) abandonaram o tratamento, 1 (0,3%) morreu por tuberculose, 2 (0,7%) morreram por outras causas, 56 (18,7%) foram transferidos de local de tratamento e 2 (0,7%) desenvolveram TB drogarresistente.

Esses casos foram avaliados quanto às suas características individuais, clínicas e institucionais (Tabela 1) e categorizados de acordo com a situação de encerramento do tratamento (sucesso e insucesso).

**Tabela 1:** Características individuais e clínicas dos casos notificados com tuberculose na população privada de liberdade do Espírito Santo, no período de 2014 a 2016, de acordo com a situação de encerramento do tratamento (n = 300).

Características	Sucesso n (%)	Insucesso n (%)	Total dos casos n (%)
Condições individuais			
Sexo			
Masculino	216 (73,7)	77 (26,3)	293 (97,7)
Feminino	2 (28,6)	5 (71,4)	7 (2,3)
Faixa etária			
18 a 29 anos	144 (75,0)	48 (25,0)	192 (64,0)
30 a 39 anos	53 (66,2)	27 (33,8)	80 (26,7)
40 anos ou mais	21 (75,0)	7 (25,0)	28 (9,3)
Raça/cor			
Branca	46 (65,7)	24 (34,3)	70 (23,3)
Preta	33 (70,2)	14 (29,8)	47 (15,7)
Amarela	1 (100,0)	0 (0,0)	1 (0,3)
Parda	127 (76,5)	39 (23,5)	166 (55,4)
Indígena	0 (0,0)	0 (0,0)	0 (0,0)
*Missing*	11 (68,8)	5 (31,2)	16 (5,3)
Escolaridade			
Analfabeto	6 (85,7)	1 (14,3)	7 (2,3)
1 a 4 anos	35 (56,7)	27 (43,5)	62 (20,7)
5 a 8 anos	109 (83,8)	21 (16,2)	130 (43,3)
> 8 anos	41 (78,8)	11 (21,2)	52 (17,3)
Não se aplica	1 (50,0)	1 (50,0)	2 (0,7)
*Missing*	26 (55,3)	21 (44,7)	47 (15,7)
Condições clínicas			
Aids			
Não	193 (78,1)	54 (21,9)	247 (82,3)
Sim	11 (61,1)	7 (38,9)	18 (6,0)
*Missing*	14 (40,0)	21 (60,0)	35 (11,7)
Diabetes mellitus			
Não	207 (74,2)	72 (25,8)	279 (93,0)
Sim	5 (83,3)	1 (16,7)	6 (2,0)
*Missing*	6 (40,0)	9 (60,0)	15 (5,0)
Doença mental			
Não	213 (74,2)	74 (25,8)	287 (95,7)
Sim	2 (50,0)	2 (50,0)	4 (1,3)
*Missing*	3 (33,3)	6 (66,7)	9 (3,0)
Alcoolismo			
Não	164 (74,2)	57 (25,8)	221 (73,7)
Sim	52 (72,2)	20 (27,8)	72 (24,0)
*Missing*	2 (28,6)	5 (71,4)	7 (2,3)
Entrada			
Caso novo	159 (74,0)	56 (26,0)	215 (71,7)
Recidiva	15 (65,2)	8 (34,8)	23 (7,7)
Reingresso após abandono	25 (75,8)	8 (24,2)	33 (11,0)
Não sabe	1 (100,0)	0 (0,0)	1 (0,3)
Transferência	18 (64,3)	10 (35,7)	28 (9,3)
Pós-óbito	0 (0,0)	0 (0,0)	0 (0,0)
Forma			
Pulmonar	197 (74,3)	68 (25,7)	265 (88,3)
Extrapulmonar	16 (59,3)	11 (40,7)	27 (9,0)
Pulmonar + extrapulmonar	5 (62,5)	3 (37,5)	8 (2,7)
Radiografia			
Normal	6 (66,7)	3 (33,3)	9 (3,0)
Suspeita	183 (75,0)	61 (25,0)	244 (81,3)
Outra patologia	2 (40,0)	3 (60,0)	5 (1,7)
Não realizada	20 (60,6)	13 (39,4)	33 (11,0)
*Missing*	7 (77,8)	2 (22,2)	9 (3,0)
Baciloscopia do escarro			
Negativa	56 (77,8)	16 (22,2)	72 (24,0)
Positiva	150 (72,5)	57 (27,5)	207 (69,0)
Não realizada	10 (62,5)	6 (37,5)	16 (5,3)
Não se aplica	2 (40,0)	3 (60,0)	5 (1,7)
Cultura inicial do escarro			
Negativa	37 (71,2)	15 (28,8)	52 (17,3)
Positiva	74 (81,3)	17 (18,7)	91 (30,3)
Em andamento	57 (64,0)	32 (36,0)	89 (29,7)
Não realizada	50 (73,5)	18 (23,5)	68 (22,7)
Tratamento supervisionado			
Não	21 (58,3)	15 (41,7)	36 (12,0)
Sim	191 (84,9)	34 (15,1)	225 (75,0)
*Missing*	6 (15,4)	33 (84,6)	39 (13,0)

Os casos foram em sua maioria homens, jovens (18 a 29 anos), da raça/cor parda e tinham de cinco a oito anos de estudo. Observou-se mais insucesso no tratamento entre as mulheres, nos indivíduos com a faixa etária de 30 a 39 anos e com um a quatro anos de estudo.

Entre as comorbidades e agravos associados, o mais prevalente foi o alcoolismo, relatado em 24% dos casos, seguido da aids (6,0%), diabetes (2,0%) e doença mental (1,2%). Nota-se que o insucesso do tratamento foi mais frequente entre os casos com aids e doença mental. O percentual de cura (sucesso) foi semelhante entre os indivíduos que faziam (72,2%) ou não uso do álcool (74,2%).

O tipo de entrada mais observado foi o caso novo (71,7%), assim como a forma pulmonar da tuberculose (88,3%). Os casos de TB extrapulmonar mostraram menos sucesso no tratamento quando comparados aos pulmonares.

Os exames de raios x foram em sua maioria suspeitos assim como os exames de baciloscopia e cultura de escarro foram positivos. Entre os que receberam o tratamento observado com medicação, observou-se um percentual de sucesso acima de 80%, enquanto este se aproximou dos 60% nos casos que não receberem a dose supervisionada.

Quanto às características institucionais, nota-se que a maioria dos casos se encontravam em estabelecimentos provisórios, administrados pela gestão estadual e com taxa de ocupação entre 100,00 e 199,99%. A avaliação da estrutura física mostrou que prevaleceram os casos que estavam em instituições construídas para a finalidade de encarceramento, com estrutura ampliada de saúde (74,7%), educação (70,0%) e visita (46,0%). Destaca-se também a presença de equipe de saúde ampliada diferenciada, composta por equipe de enfermagem, clínico geral, dentista, assistente social e psicólogo ou terapeuta ocupacional, ou psiquiatra, ou fisioterapeuta para 63,0% dos casos. A taxa de trabalho foi de 0,10 a14,99% nas unidades em que foram diagnosticados 65,6% dos casos de TB, e a taxa de educação mais predominante foi de 15,00 a 29,99%, representando 32,3% deles. Esses dados diferenciados por situação de encerramento (sucesso e insucesso) são detalhados na Tabela 2.

**Tabela 2:** Características institucionais dos casos notificados com tuberculose na população privada de liberdade do Espírito Santo, no período de 2014 a 2016, de acordo com a situação de encerramento do tratamento (n = 300).

Características	Sucesso n (%)	Insucesso n (%)	Total dos casos n (%)
Condições institucionais			
Tipo do estabelecimento			
Provisório	98 (71,0)	40 (29,0)	138 (46,0)
Fechado	94 (82,5)	20 (17,5)	114 (38,0)
Semiaberto	17 (50,0)	17 (50,0)	34 (11,3)
Misto	9 (64,3)	5 (35,7)	14 (4,7)
Medida de segurança	0 (0,0)	0 (0,0)	0 (0,0)
Taxa de ocupação			
Abaixo de 99,99%	10 (58,8)	7 (41,2)	17 (5,7)
100,00–199,99%	180 (72,6)	68 (27,4)	248 (82,6)
200,00% ou mais	28 (80,0)	7 (20,0)	35 (11,7)
Gestão			
Pública	207 (71,9)	81 (28,1)	288 (96,0)
Cogestão	11 (91,7)	1 (8,3)	12 (4,0)
Tipo de construção			
Concebida	197 (72,2)	76 (27,8)	273 (91,0)
Adaptada	21 (77,8)	6 (22,2)	27 (9,0)
Módulo visita			
Não	27 (71,1)	11 (28,9)	38 (12,7)
Mínimo	84 (67,7)	40 (32,3)	124 (41,3)
Ampliado	107 (77,5)	31 (22,5)	138 (46,0)
Módulo saúde			
Apoio	6 (66,7)	3 (33,3)	9 (3,0)
Mínimo	33 (67,3)	16 (32,7)	49 (16,3)
Mínimo diferenciado	13 (72,2)	5 (27,8)	18 (6,0)
Ampliado	166 (74,1)	58 (25,9)	224 (74,7)
Módulo educação			
Não	49 (73,1)	18 (26,9)	67 (22,3)
Mínimo	18 (78,3)	5 (21,7)	23 (7,7)
Ampliado	151 (71,9)	59 (28,1)	210 (70,0)
Equipe de saúde			
Somente técnico	10 (52,6)	9 (47,4)	19 (6,3)
Mínima	20 (76,9)	6 (23,1)	26 (8,7)
Mínima diferenciada	35 (70,0)	15 (30,0)	50 (16,7)
Ampliada	8 (50,0)	8 (50,0)	16 (5,3)
Ampliada diferenciada	145 (76,7)	44 (23,3)	189 (63,0)
Taxa de trabalho			
Não	30 (78,9)	8 (21,1)	38 (12,7)
0,10–14,99%	142 (72,1)	55 (27,9)	197 (65,6)
15,00–29,99%	35 (89,7)	4 (10,3)	39 (13,0)
30,00–44,99%	3 (27,3)	8 (72,7)	11 (3,7)
45,00–59,99%	3 (60,0)	2 (40,0)	5 (1,7)
60,00% ou mais	5 (50,0)	5 (50,0)	10 (3,3)
Taxa de educação			
Não	51 (70,8)	21 (29,2)	72 (24,0)
0,10–14,99%	61 (65,6)	32 (34,4)	93 (31,0)
15,00–29,99%	77 (79,4)	20 (20,6)	97 (32,3)
30,00–44,99%	9 (81,8)	2 (18,2)	11 (3,7)
45,00–59,99%	11 (64,7)	6 (35,3)	17 (5,7)
60,00% ou mais	9 (90,0)	1 (10,0)	10 (3,3)

A Tabela 3 aponta somente as características que permaneceram no modelo final. A análise ajustada mostrou que o tratamento de forma supervisionada para a tuberculose é um fator protetor para o desfecho de insucesso no tratamento (RC = 0,29; IC95% 0,01–0,76).

**Tabela 3:** Análise bruta e ajustada (hierarquizada) dos casos notificados de tuberculose na população privada de liberdade do Espírito Santo, no período de 2014 a 2016, de acordo com a situação de encerramento do tratamento (n = 300).

Características	RC bruta (IC95%)	p-valor	RC ajustada (IC95%)	P-valor
				
Aids		0,105		0,159
Não	1,00		1,00	
Sim	2,27 (0,80–6,06)		2,26 (0,59–8,62)	
Forma		0,112		0,044
Pulmonar	1,00		1,00	
Extrapulmonar	1,99 (0,86–4,46)		1,81 (0,38–8,52)	
Pulmonar + Extrapulmonar	1,73 (0,34–7,27)		4,98 (0,51–48,11)	
Baciloscopia do escarro		0,058		0,094
Negativa	1,00		1,00	
Positiva	1,33 (0,71–2,56)		1,31 (0,42–4,40)	
Não realizada	2,10 (0,63–6,59)		2,19 (0,54–16,19)	
Não se aplica	5,25 (0,80–42,54)		43,85 (1,47–1303,20)	
Radiografia		0,084		0,220
Normal	1,00		1,00	
Suspeita	0,66 (0,17–3,23)		0,38 (0,04–3,08)	
Outra patologia	3,00 (0,32–34,76)		1,29 (0,07–22,38)	
Não realizada	1,30 (0,28–7,02)		0,92 (0,09–8,93)	
Tratamento supervisionado		< 0,001		0,002
Não	1,00		1,00	
Sim	0,24 (0,11–0,53)		0,29 (0,10–0,76)	
Gestão		0,165		0,123
Pública	1,00		1,00	
Cogestão	0,23 (0,01–1,22)		0,21 (0,26–1,70)	
Taxa de ocupação		0,124		0,037
abaixo de 99,99%	1,00		1,00	
100,00–199,99%	0,53 (0,19–1,53)		0,51 (0,18–1,43)	
200,00% ou mais	0,35 (0,09–1,27)		0,23 (0,06–0,88)	
Módulo visita		0,170		0,040
Não	1,00		1,00	
Mínimo	1,16 (0,53–2,67)		1,03 (0,45–2,33)	
Ampliado	0,71 (0,32–164)		0,52 (0,22–1,23)	

RC: razão de chances; IC95%: intervalo de confiança de 95%

## DISCUSSÃO

Este estudo apresentou algumas limitações. Não foram incluídas variáveis que permitissem conhecer o tempo de privação de liberdade no momento do diagnóstico ou relativas à história pregressa de encarceramento, que poderiam influenciar principalmente no diagnóstico da doença, apesar de ainda serem escassos dados que mostrem alguma associação com o desfecho do tratamento. Além disso, pode estar subestimado o impacto da TB na PPL na carga geral da doença quando consideradas as situações em que os indivíduos a desenvolvem após sua soltura, embora tenham adquirido o bacilo durante o encarceramento. Sugere-se que sejam realizados outros estudos, que sejam capazes de avaliar essas questões.

No período estudado, o CTV apresentou a maior taxa de casos diagnosticados para TB, com 17,3 por 1.000 indivíduos privados de liberdade. Destaca-se que o CTV possui características diferenciadas em relação às demais instituições, por se tratar de uma porta de entrada para o sistema prisional do ES e por isso apresentar altíssima rotatividade, com uma média de 690 ingressos a cada mês^7^. O fluxo estabelecido prevê que esses ingressantes permaneçam nessa unidade pelo prazo máximo de 48 horas até que seja realizada sua transferência, de acordo com perfil e situação jurídica, identificados por meio de um cadastro inicial. No entanto, esse prazo não é cumprido, sendo observada a permanência de presos por até 30 dias^8^.

Segundo o Protocolo de Controle de Tuberculose Pulmonar da População Prisional do ES, baseado nas diretrizes do MS, deve ser realizada a busca ativa de sintomáticos respiratórios por meio de um formulário de triagem específico aplicado no ingresso no sistema prisional^9^, o que possivelmente contribuiu para a identificação do número superior de casos de TB no CTV comparado com as demais unidades. Além disso, quando esses indivíduos são transferidos para outras unidades, já foram triados inicialmente para TB. No entanto, apesar de o diagnóstico ser realizado durante o encarceramento, é importante refletir sobre o mecanismo de transmissão do bacilo da TB e seu tempo de incubação. A partir dos dados deste estudo, não é possível afirmar que a unidade prisional foi o local de contágio da TB, já que a pessoa privada de liberdade poderia já ser portadora do bacilo antes do encarceramento e ter apenas progredido para a doença ativa durante esse período. Dessa forma, embora não seja a fonte, o ambiente prisional teria contribuído para a evolução da doença^3^. Estudos genéticos poderiam fornecer uma melhor compreensão da dinâmica da transmissão de *M. tuberculosis* e esclarecer essa questão. O conhecimento das formas em que o microrganismo está disperso na população intra e extramuros é importante para as estratégias de prevenção e controle da doença^10^.

Os casos de TB registrados na PPL do ES nos anos de 2014, 2015 e 2016 representaram respectivamente 7,5%, 6,1% e 7,8% do total de casos notificados. Em 2014, 8,4% do total de casos no Brasil foram encontrados entre a PPL, embora esse grupo representasse aproximadamente 0,3% da população total do país nesse ano^11^.

Segundo o MS, dados comparativos da situação de encerramento do tratamento da TB em 2015 mostraram para a PPL taxas de cura, abandono e transferência de 73,2%, 7,7% e 5,5%, enquanto na população em geral essas taxas foram de 71,0%, 9,8% e 5,0%, respectivamente^12^. As taxas de cura (72,6%) e abandono (7,0%) foram semelhantes às observadas neste estudo, porém os índices de transferência (18,7%) foram superiores, o que pode ser explicado pela logística adotada pela Secretaria de Justiça do ES, que elegeu uma unidade prisional como porta de entrada para o sistema penitenciário, a partir da qual é realizada a alocação para as outras unidades.

As frequentes transferências entre as unidades prisionais são uma importante causa de perda de seguimento; dessa forma, a comunicação entre os profissionais de segurança e saúde deve ser estabelecida de forma ágil e constante, visando a minimizar os casos de abandono de tratamento. É essencial que o prontuário de saúde acompanhe o paciente nas transferências entre as unidades prisionais. Além disso, o MS destaca que a mobilidade no sistema prisional aumenta o risco de adoecer por TB, haja vista a circulação entre as diferentes instituições penitenciárias, serviços de saúde e comunidade geral, durante e após o encarceramento^13^. Embora a taxa de mulheres presas tenha aumentado 10,7% ao ano de 2005 a 2014, os homens ainda são a grande maioria da PPL, sendo a média brasileira de 5,8% mulheres presas para 94,2% de homens^5^. No ES, os homens representaram 93,6% do total de pessoas presas em 2014^5^, corroborando o alto percentual de casos de TB no sexo masculino observado neste estudo. No entanto, apesar de serem minoria, a pesquisa mostrou uma proporção maior de insucesso do tratamento nas mulheres (71,4%) que nos homens (26,3%). Uma pesquisa que avaliou a TB na PPL no Brasil de 2009 a 2014^11^ mostrou que as mulheres privadas de liberdade apresentaram taxa de coinfecção pelo HIV (24,1%) superior à taxa correspondente entre homens (15,2%). Neste estudo a coinfecção estava presente em 28,6% das mulheres e em 5,5% dos homens, podendo contribuir de forma importante para o cenário de insucesso observado, ainda que associada a outras características.

Dados do Infopen mostram que aproximadamente 55% da PPL no Brasil tem de 18 a 29 anos de idade^6^, sendo essa a faixa etária em que mais se registraram casos de TB no estudo (64%). Considerando a faixa etária mais ampla, de 18 a 39 anos, observam-se 90,7% dos casos registrados. Estudos anteriores mostram também o registro da maioria dos casos de TB na PPL na faixa etária de 18 a 39 anos (74,7%), embora apenas 39,8% dos casos de TB sejam registrados nessa faixa etária na população em geral^11^. No ano de 2014, 25,6% dos casos de TB em homens de 20 a 29 anos registrados no Brasil estavam privados de liberdade^11^.

A tuberculose extrapulmonar, observada em 9,0% do total de casos na PPL do ES, mostrou-se semelhante à registrada no país, de 8,2%^11^. Os resultados apontaram um percentual maior de sucesso do tratamento para a forma pulmonar (74,3%) que para a extrapulmonar (59,3%). A realização do diagnóstico da TB extrapulmonar apresenta um grau mais elevado de dificuldade, visto que algumas formas apresentam sinais e sintomas inespecíficos e exigem exames diferenciados de acesso restrito à PPL, com consequente atraso do início do tratamento, podendo explicar esse achado^13^.

A descrição institucional observada refere-se às características das unidades prisionais de localização do caso de TB no momento do diagnóstico e reflete o contexto prisional vivenciado no ES de uma maneira geral. Em 2016, a taxa de ocupação dos estabelecimentos prisionais no país era 197,4% e no ES era 144,7%, enquanto a taxa de presos sem condenação no Brasil era de 40,2% e de 42,3% no ES^14^, de acordo com o observado na maioria dos casos notificados no estudo.

No ano de 2006, o sistema penitenciário do ES viveu superlotação, rebeliões violentas, homicídios e fugas, gerando a ausência de uma estrutura mínima que possibilitasse a gestão da segurança ou política de ressocialização. Sendo assim, em 2009, houve uma intervenção federal realizada pelo Conselho Nacional de Política Criminal e Penitenciária, o que motivou uma restruturação no sistema prisional do ES por meio da construção de forma padronizada de 26 unidades prisionais, que incluíam estrutura física para visita, educação, trabalho e saúde. A gestão estadual objetivou implementar políticas nas áreas de segurança e ressocialização, abrangendo assistência à saúde, oferta de estudo formal, capacitação e trabalho^15^. O ES atualmente apresenta índices de trabalho e educação entre os melhores registrados no país^15,14,16^. A construção de unidades ventiladas e iluminadas contribui de forma benéfica para a redução da transmissão de infecções respiratórias, conforme tratado pelo Manual de Intervenções Ambientais para o Controle da Tuberculose nas Prisões, que apresenta soluções de melhoria das condições ambientais para a preservação da saúde dos que frequentam o espaço prisional sem comprometer a segurança^17^.

No que tange à saúde, a cobertura do Programa de Saúde Prisional no ES era de 75,2% em 2014^18^, sendo ampliada para 100,0% no ano de 2018. Este estudo mostrou a importância da atuação da equipe de saúde para o sucesso do tratamento. A PNAISP, instituída pelo Ministério da Saúde em 2014, objetiva garantir o acesso das pessoas privadas de liberdade ao cuidado integral no SUS, como ponto de atenção da Rede de Atenção à Saúde (RAS). A PNAISP e suas portarias normatizam os tipos de equipes e financiamento dos recursos. A Equipe de Atenção Básica Prisional (EABp) apresenta composição multiprofissional, e sua modalidade e carga horária de atuação são determinadas pelo número de pessoas custodiadas e o perfil epidemiológico das unidades^4^.

A realização do tratamento diretamente observado (TDO) mostrou-se associada ao sucesso do tratamento, reafirmando o efeito benéfico do TDO já demostrado em outros estudos^19^. Uma pesquisa mostrou que o tratamento supervisionado reduz em 25% os desfechos desfavoráveis do tratamento da tuberculose, e que pacientes que não receberam o TDO foram mais propensos ao abandono do tratamento (RC = 0,62; IC95% 0,57–0,66), óbito por TB (RC = 0,68; IC95% 0,61–0,77) e resultados de tratamento desconhecidos (RC = 0,71; IC95% 0,66–0,76)^20^. O TDO é preconizado pelo MS para a PPL, destacando como benefícios o fortalecimento do vínculo com o profissional de saúde, a garantia de acesso rápido ao serviço em caso de efeitos adversos pelo tratamento, o estabelecimento de barreiras para que a medicação seja usada como elemento de troca e pressão, a oferta de troca de informações e estabelecimento do cuidado, entre outros^13^.

Além dos aspectos já discutidos no artigo, o MS lista como principais obstáculos para a implementação de estratégias de controle no ambiente prisional: o desconhecimento sobre a TB pela PPL e profissionais de segurança que atuam nas prisões; a subvalorização dos sintomas da doença pela PPL, dificultando o diagnóstico precoce; a dificuldade de acesso da PPL aos serviços de saúde, assim como a escassez de recursos humanos e financeiros destinados a esses serviços; a baixa adesão da PPL ao tratamento e ações de prevenção; e a estigmatização e segregação geradas pela TB, considerando a importância da proteção pelo pertencimento ao grupo^13^.

Apesar dos desafios, o estudo mostrou que é de extrema importância buscar conhecer quais componentes influenciam no desfecho do tratamento da tuberculose na PPL, com vistas à implementação de ações que proporcionem a cura do tratamento e reduzam índices de insucesso. Conhecer as características que levam principalmente ao abandono contribui para interromper a cadeia de transmissão da doença e para sua prevenção, não somente entre a PPL, mas entre o grupo de familiares e trabalhadores que convivem com o ambiente prisional.
